# Insights into the functional mechanisms of three terpene synthases from *Lavandula angustifolia* (Lavender)

**DOI:** 10.3389/fpls.2024.1497345

**Published:** 2024-12-03

**Authors:** Dafeng Liu, Hongying Song, Huashui Deng, Ablikim Abdiriyim, Lvxia Zhang, Ziwei Jiao, Xueru Li, Lu Liu, Shuangqin Bai

**Affiliations:** ^1^ Xinjiang Key Laboratory of Lavender Conservation and Utilization, College of Biological Sciences and Technology, Yili Normal University, Yining, Xinjiang, China; ^2^ School of Life Sciences, Xiamen University, Xiamen, Fujian, China

**Keywords:** lavender, *Lavandula angustifolia*, terpene synthases, gene expression, enzymatic activity assay

## Abstract

Lavender species are of significant economic value being cultivated extensively worldwide for their essential oils (EOs), which include terpenes that play crucial roles in the cosmetic, personal care, and pharmaceutical industries. The terpene synthases in lavender, such as *Lavandula angustifolia* linalool synthase (LaLINS), limonene synthase (LaLIMS), and bergamotene synthase (LaBERS), are key enzymes in terpene biosynthesis. However, the functional mechanisms underlying these enzymes remain poorly understood. Here, we used AlphaFold2 to predict the three-dimensional structures of LaLINS, LaLIMS, and LaBERS. The hydrodynamic radii of LaLINS, LaLIMS, and LaBERS were 5.7 ± 0.2, 6.2 ± 0.3, and 5.4 ± 0.2 nm, respectively. Mutations D320A or D324A led to a complete loss of activity in LaLINS compared to the wild-type (WT) enzyme; similarly, mutations D356A or D360A abolished activity in LaLIMS, and D291A or D295A eliminated activity in LaBERS. Furthermore, the genes *LaLINS*, *LaLIMS*, and *LaBERS* exhibited significantly higher expression levels in leaves compared to stems and flowers, with peak expression occurring at 8:00 a.m. Our findings contribute to a deeper understanding of terpene biosynthesis in lavender and offer insights for improving essential oil production through genetic engineering.

## Introduction

Lavenders are small, aromatic shrubs cultivated worldwide for their essential oils (EOs), which consist of a complex mixture of mono- and sesquiterpenoid alcohols, esters, oxides, and ketones. The genus lavender encompasses 30 known species, with three species of particular economic significance: *Lavandula angustifolia*, *Lavandula latifolia*, and *Lavandula x intermedia*, the latter being a natural hybrid between *Lavandula latifolia* and *L. angustifolia* (Crişan et al., 2023; De Melo Alves Silva et al., 2023). Notably, the highest-quality essential oils are distilled from the flowering tips of *L. angustifolia*, commonly referred to as “true lavender,” which has been valued for its distinctive fragrance since ancient times (Landmann et al., 2007; Crişan et al., 2023; De Melo Alves Silva et al., 2023). Lavender essential oils are extensively used in the production of cosmetics, personal hygiene products, and alternative medicines (Hedayati et al., 2024; Khan et al., 2024; Li et al., 2024). For example, essential oils with elevated camphor content are employed in inhalants to relieve coughs and colds and serve as active components in liniments and balms used as topical analgesics (Malloggi et al., 2021; Batiha et al., 2023; Braunstein and Braunstein, 2023). Moreover, camphor has been explored as a potential radiosensitizing agent and has been applied to oxygenate tumors prior to radiotherapy (Bungau et al., 2023; Crişan et al., 2023; De Melo Alves Silva et al., 2023; Dewanjee et al., 2023; Khan et al., 2024).

Lavender essential oils exhibit diverse terpene compositions, primarily comprising monoterpenes and sesquiterpenes. Across various lavender species, approximately 50–60 monoterpenes have been identified, although only a select few dominate the characteristic EOs of each species. The most common monoterpenes in lavenders include linalool, linalool acetate, borneol, camphor, and 1,8-cineole (Mitra et al., 2023; Dold et al., 2024). Of note, camphor, linalool, and linalool acetate are key determinants of lavender EO quality. Essential oils with a higher ratio of linalool and linalool acetate to camphor are regarded as “high quality” (Malloggi et al., 2021; Batiha et al., 2023; Hedayati et al., 2024). Although these terpenes are the primary bioactive and aromatic constituents, their biosynthetic pathways have not been thoroughly studied. Understanding these pathways, along with the identification of key terpene synthases, such as *L. angustifolia* linalool synthase (LaLINS), limonene synthase (LaLIMS), and bergamotene synthase (LaBERS), provides a valuable toolkit of natural catalysts. These insights are instrumental in developing enhanced lavender chemotypes and optimizing terpene production through chemoenzymatic methods and metabolic engineering.

Herein, we found that the mutations D320A or D324A resulted in a complete loss of activity in LaLINS compared to the wild-type (WT) protein. Similarly, the mutations D356A or D360A fully abolished the activity of LaLIMS, and D291A or D295A led to a total loss of activity in LaBERS. Furthermore, the expression of the *LaLINS*, *LaLIMS*, and *LaBERS* genes was significantly higher in leaves compared to stems and flowers, with peak expression observed at 8:00 a.m. Our results elucidate the functional mechanisms of the three terpene synthases (LaLINS, LaLIMS, and LaBERS) in lavender offering valuable insights for improving the quality of lavender essential oils through genetic engineering. This knowledge may also inform the development of cosmetic and personal care products, as well as alternative medicines.

## Results

### Bioinformatics analysis

**Figure 1:**
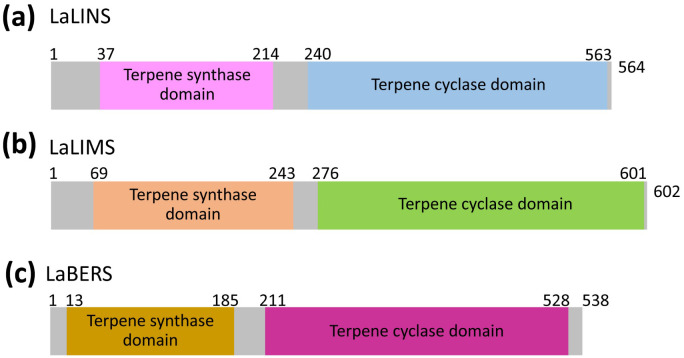
Organization of clusters of three terpene synthases. **(A)** For LaLINS, **(B)** for LaLIMS, and **(C)** for LaBERS. Schematic representation of terpene synthase domain and terpene cyclase domain.

*L. angustifolia* linalool synthase (LaLINS), limonene synthase (LaLIMS), and bergamotene synthase (LaBERS) are terpene synthases characterized by the presence of the following two conserved domains: the terpene synthase domain and the terpene cyclase domain (**Figure 1**; **Supplementary Figure S1**). The molecular weights of LaLINS, LaLIMS, and LaBERS were approximately 65.65, 70.35, and 62.41 kDa, respectively. Their molecular formulas were determined to be C_2979_H_4528_N_772_O_874_S_16_ for LaLINS, C_3167_H_4903_N_847_O_927_S_20_ for LaLIMS, and C_2798_H_4318_N_732_O_831_S_28_ for LaBERS. The isoelectric points (pI) were calculated as 5.08 for LaLINS, 5.49 for LaLIMS, and 5.16 for LaBERS, with corresponding protein instability indices of 48.94, 43.56, and 45.59, respectively.

### Prediction and quality assessment of structural models of the three terpene synthases

**Figure 2:**
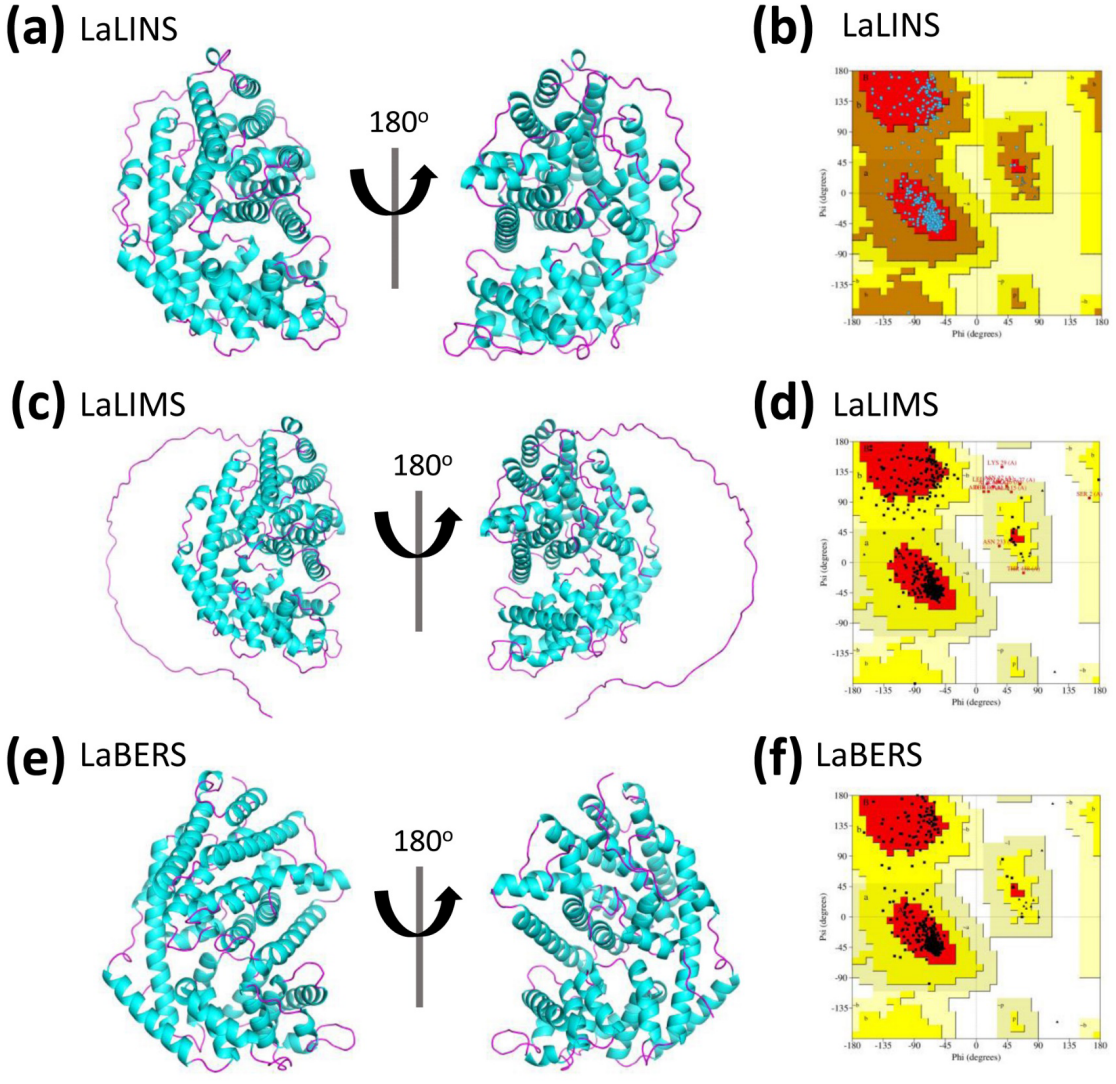
Prediction and quality assessment of structural model of three terpene synthases. **(A, C, E)** The three-dimensional (3D) structures are depicted in ribbon representation from two different orientations, with helices and sheets colored pink and cyan, respectively. These 3D structures were predicted using AlphaFold2. **(B, D, F)** Ramachandran Plot analysis was conducted for structural validation, with the most favorable regions highlighted in red and less favorable regions depicted in progressively lighter shades.

The three-dimensional (3D) structures of LaLINS, LaLIMS, and LaBERS were predicted using Alphafold2 (Jumper et al., 2021; Tunyasuvunakool et al., 2021; Wayment-Steele et al., 2023) (**Figure 2**). Unlike conventional homology modeling techniques, Alphafold2 utilizes advanced deep learning algorithms, which significantly improve the accuracy and reliability of protein structure predictions (**Supplementary Figures S2–S5**).

To assess the predicted structural models, the Ramachandran Plot was used to assess the dihedral angles of the protein backbone ensuring the conformational integrity of the proteins. The structural models of LaLINS, LaLIMS, and LaBERS demonstrated that 93.6%, 88.2%, and 93.9% of their residues, respectively, were located within the most favored regions (**Figures 2B, D, F**; **Table 1**). Additionally, 6.4%, 9.7%, and 6.1% of residues were found in the allowed regions, while 0%, 1.4%, and 0% were situated in the generously allowed regions (**Figures 2B, D, F**; **Table 1**). These findings indicate that the structural models of the three enzymes are of high quality.

**Table 1:** Ramchandran plot analysis of structural models of the three terpene synthases using PDBsum.

Protein	Residues in most favored regions*^a^*		Residues in additional allowed regions		Residues in generously allowed regions		Residues in disallowed regions	
	Number of residues	% of residues*^a^*	Number of residues	% of residues	Number of residues	% of residues	Number of residues	% of residues
LaLINS*^b^*	483	93.6	33	6.4	0	0	0	0
LaLIMS*^c^*	493	88.2	54	9.7	8	1.4	4	0.7
LaBERS*^d^*	464	93.9	30	6.1	0	0	0	0

*^a^* A good quality model is expected to have over 90% residues in most favored regions; *^b^* Number of end residues (excl. Gly and Pro): 2; *^b^* Number of glycine residues (shown as triangles): 28; *^b^* Number of proline residues: 18. *^c^* Number of end residues (excl. Gly and Pro): 1; *^c^* Number of glycine residues (shown as triangles): 20; *^c^* Number of proline residues: 22. *^d^* Number of end residues (excl. Gly and Pro): 1; *^d^* Number of glycine residues (shown as triangles): 25; *^d^* Number of proline residues: 18.

### Characterization of the three terpene synthases by dynamic light scattering

**Figure 3:**
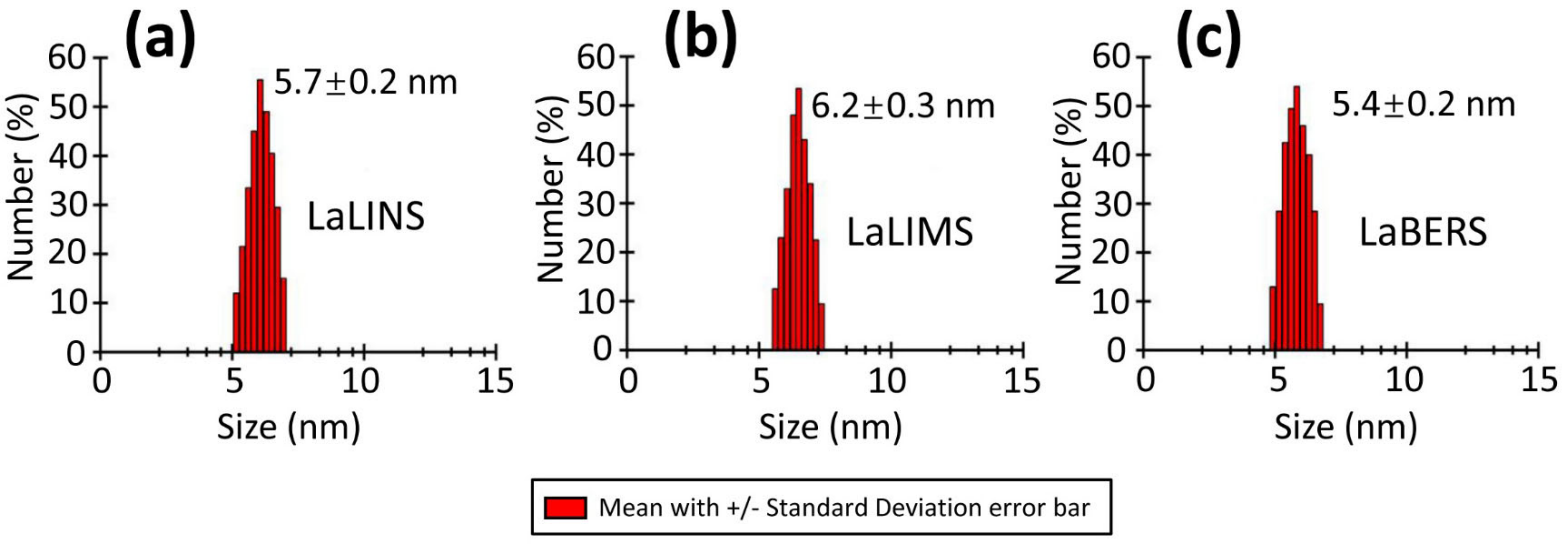
Dynamic light scattering (DLS) spectrum of three terpene synthases. **(A)** LaLINS, **(B)** LaLIMS, and **(C)** LaBERS demonstrated hydrodynamic radii of 5.7 ± 0.2, 6.2 ± 0.3, and 5.4 ± 0.2 nm, respectively, indicating that the three terpene synthases exist in their monomeric forms.

To investigate the oligomeric state of the three terpene synthases (LaLINS, LaLIMS, and LaBERS), dynamic light scattering (DLS) experiments were performed following centrifugation to measure their hydrodynamic radii. The results revealed hydrodynamic radii of 5.7 ± 0.2 nm for LaLINS, 6.2 ± 0.3 nm for LaLIMS, and 5.4 ± 0.2 nm for LaBERS (**Figure 3**) showing that all three terpene synthases exist in monomeric forms.

### Enzymatic activity of the three terpene synthases

**Figure 4:**
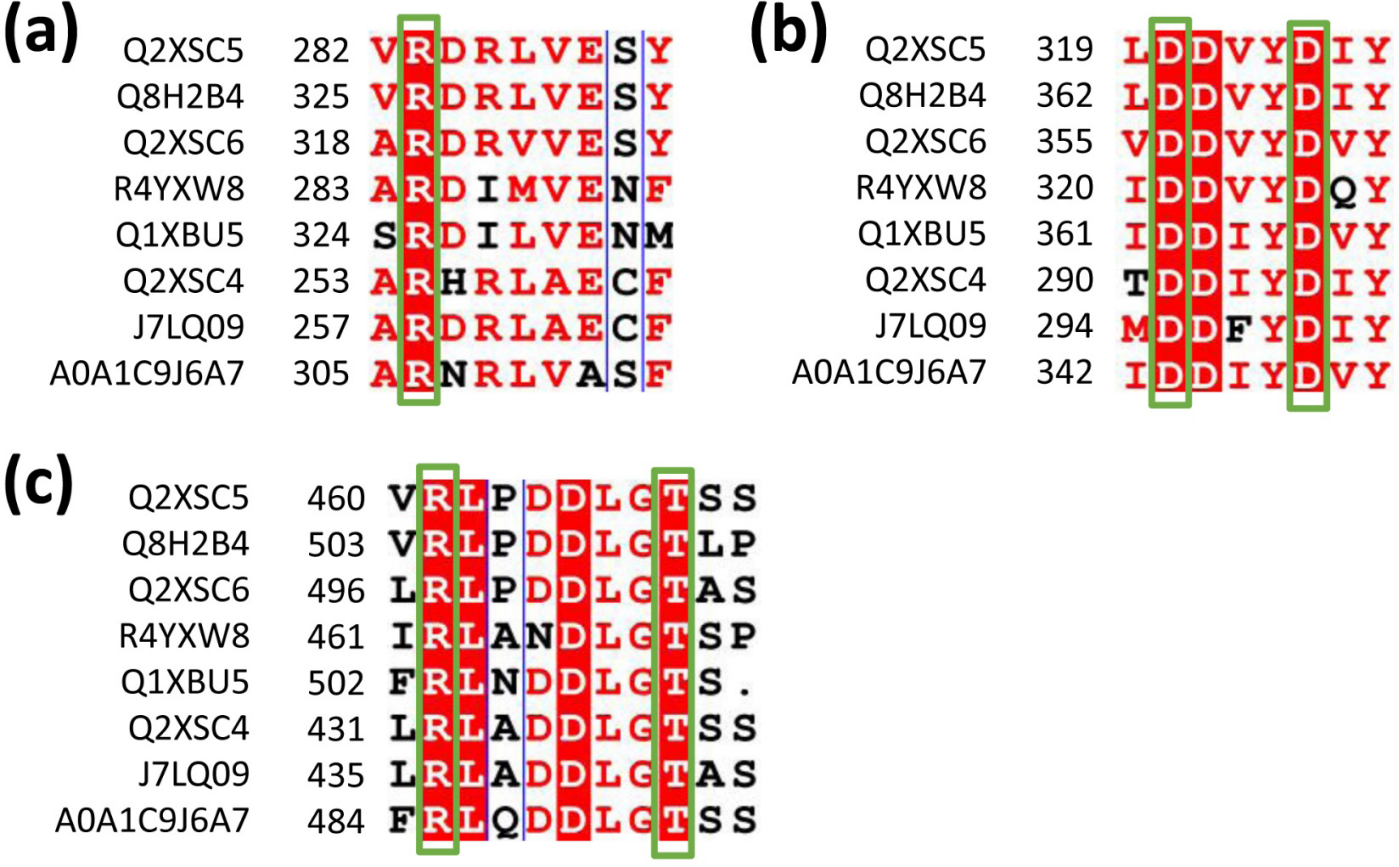
Sequence alignment of conserved residues from various species.The conserved amino acids in LaLINS are identified as: **(A)** R283, **(B)** D320 and D324, and **(C)** R461 and T468. In LaLIMS, the conserved amino acids are: **(A)** R319, **(B)** D356 and D360, and **(C)** R497 and T504. For LaBERS, the conserved amino acids include: **(A)** R254, **(B)** D291 and D295, and **(C)** R432 and T439. Q2XSC5, Lavandula angustifolia (Lavender); Q8H2B4, Mentha aquatica (Water mint); Q2XSC6, Lavandula angustifolia (Lavender); R4YXW8, Coffea arabica (Arabian coffee); Q1XBU5, Solanum lycopersicum (Tomato, Lycopersicon esculentum); Q2XSC4, Lavandula angustifolia (Lavender); J7LQ09, Phyla dulcis (Aztec sweet herb, Lippia dulcis); and A0A1C9J6A7, Citrus sinensis (Sweet orange, Citrus aurantium var. Sinensis).

**Figure 5:**
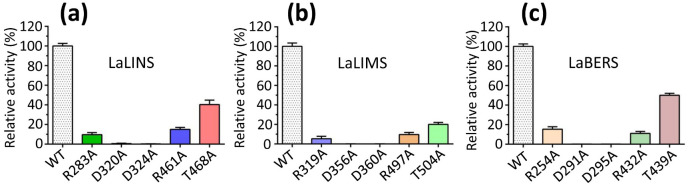
Enzymatic characterization of three terpene synthases. The activities of the wild-type (WT) proteins and specific mutants were quantitatively assessed. **(A)** Mutations R283A, R461A, and T468A caused a significant decrease in activity compared to WT LaLINS, whereas mutations D320A and D324A led to complete loss of activity. **(B)** Similarly, R319A, R497A, and T504A markedly reduced the activity of LaLIMS compared to WT protein, while D356A and D360A resulted in total inactivity. **(C)** For LaBERS, mutations R254A, R432A, and T439A dramatically decreased the activity relative to WT protein, with D291A and D295A completely abolishing activity. The activity of the corresponding wild-type (WT) protein was set to 100%.

Based on the sequence alignments of terpene synthases (**Figure 4**; **Supplementary Figure S1**), targeted mutation experiments were conducted. The results showed that the mutations R283A, R461A, and T468A in LaLINS resulted in a two- to fivefold decrease in activity compared to the wild-type (WT) protein, whereas the mutations D320A and D324A completely abolished the activity (**Figures 4**, **5A**). Similarly, the mutations R319A, R497A, and T504A in LaLIMS led to a 5- to 20-fold reduction in the activity, while D356A and D360A entirely eliminated its activity (**Figures 4**, **5B**). For LaBERS, the mutations R254A, R432A, and T439A caused a two- to sevenfold decrease in the activity, with D291A and D295A resulting in a complete loss of function (**Figures 4**, **5C**). These findings highlight the critical role of negatively charged aspartic acid residues in protein function, particularly their proximity to the substrate, which underscores the importance of these mutations. The substrate likely fits within the binding pocket of the terpene cyclase domain through electrostatic interactions involving magnesium ions (Mg^2+^) and the side chains of aspartic acids. These results demonstrated that conserved negatively charged sites are essential for the terpene synthase activity of LaLINS, LaLIMS, and LaBERS, consistent with previous studies (Starks et al., 1997; Noel et al., 2010).

### Gene expression profiles of the three terpene synthases in different tissues

**Figure 6:**
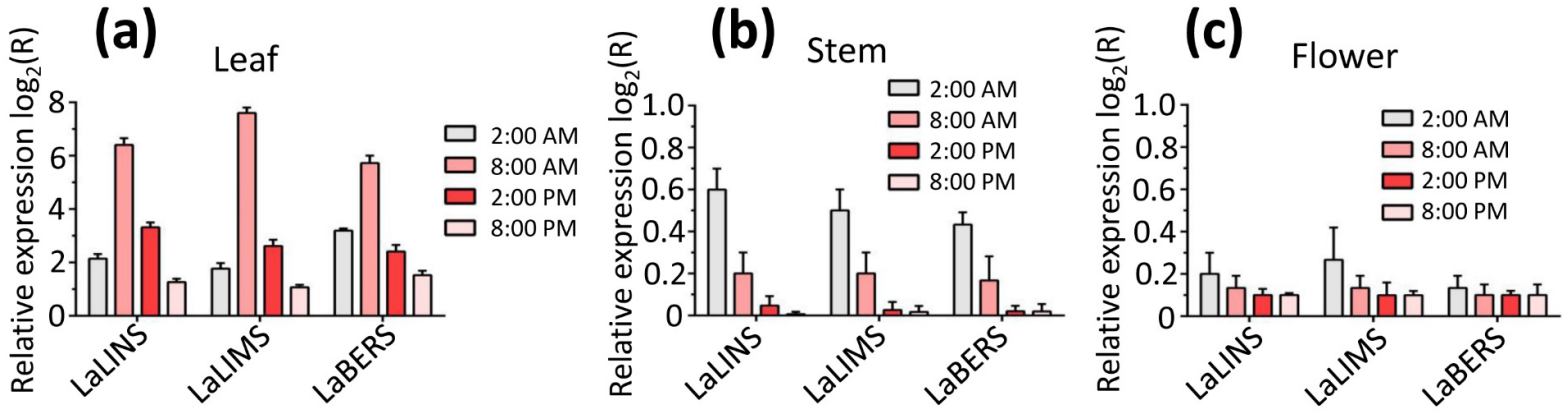
Expression levels of three terpene synthases in **(A)** leaves, **(B)** stems, and **(C)** flowers within a 24-h day/night cycle. Relative expression analysis was conducted *via* RT-qPCR, with beta-actin gene serving as the housekeeping gene. Data were analyzed using the 2^−ΔΔCT^ method.

To evaluate the expression levels of the three terpene synthase genes (*LaLINS*, *LaLIMS*, and *LaBERS*) and the corresponding accumulation of metabolites at various times and in different tissues, real-time quantitative polymerase chain reaction (RT-qPCR) analysis was conducted using gene-specific primers (**Supplementary Table S1**). We found that the expression of *LaLINS*, *LaLIMS*, and *LaBERS* was significantly higher in leaves compared to that in stems and flowers, with peak expression occurring at 8:00 a.m. (**Figure 6**). These findings suggested that certain terpenes associated with *LaLINS*, *LaLIMS*, and *LaBERS* are synthesized and emitted from specific plant tissues at particular times, corresponding to the spatiotemporal expression patterns of their respective terpene synthases (**Figure 6**). This implied that terpenoid biosynthesis is regulated at the transcriptional level. Leaf and stem tissues were primarily associated with secretory structures that produced large quantities of terpenes, predominantly monoterpenes and sesquiterpenes.

To further explore the spatiotemporal patterns of gene expression, gas chromatography-mass spectrometry (GC-MS) was employed to analyze the metabolites produced by the three terpene synthases. The results indicated that the highest metabolite yield occurred in the leaves compared to the corresponding yields from the other tissues (stem and flower) (**Table 2**). Furthermore, the metabolite yield from the three terpene synthases at 8:00 a.m. was significantly greater than that observed at other time points (2:00 a.m., 2:00 p.m., and 8:00 p.m.) (**Table 2**). These findings are consistent with the results obtained from RT-qPCR analysis.

**Table 2:** Analysis of metabolite resulting from three terpene synthases.

Tissue	The three terpene synthases	Time	Metabolite (quantity μg/g dry tissue)
Leaf	LaLINS	2:00 a.m.	35.27 ± 1.10
		8:00 a.m.	119.23 ± 2.31
		2:00 p.m.	42.81 ± 1.19
		8:00 p.m.	21.38 ± 2.45
	LaLIMS	2:00 a.m.	38.67 ± 2.38
		8:00 a.m.	126.19 ± 1.32
		2:00 p.m.	41.39 ± 2.38
		8:00 p.m.	21.86 ± 1.57
	LaBERS	2:00 a.m.	43.26 ± 2.39
		8:00 a.m.	107.84 ± 2.36
		2:00 p.m.	40.17 ± 2.26
		8:00 p.m.	24.49 ± 1.92
Stem	LaLINS	2:00 a.m.	2.12 ± 0.15
		8:00 a.m.	2.09 ± 0.78
		2:00 p.m.	0.00
		8:00 p.m.	0.00
	LaLIMS	2:00 a.m.	2.78 ± 0.39
		8:00 a.m.	2.67 ± 0.83
		2:00 p.m.	0.00
		8:00 p.m.	0.00
	LaBERS	2:00 a.m.	2.24 ± 0.58
		8:00 a.m.	2.91 ± 0.62
		2:00 p.m.	0.00
		8:00 p.m.	0.00
Flower	LaLINS	2:00 a.m.	1.10 ± 0.23
		8:00 a.m.	0.98 ± 0.37
		2:00 p.m.	0.79 ± 0.16
		8:00 p.m.	0.89 ± 0.37
	LaLIMS	2:00 a.m.	0.95 ± 0.24
		8:00 a.m.	1.02 ± 0.37
		2:00 p.m.	0.87 ± 0.26
		8:00 p.m.	0.62 ± 0.34
	LaBERS	2:00 a.m.	0.79 ± 0.12
		8:00 a.m.	0.81 ± 0.23
		2:00 p.m.	0.93 ± 0.11
		8:00 p.m.	0.88 ± 0.24

These results are consistent with previous studies indicating that some terpene synthases show preferential expression in leaves (Dornelas and Mazzafera, 2007; Hamberger et al., 2020).

## Discussion

In this work, we observed that mutations R283A, R461A, or T468A significantly diminished the activity of LaLINS compared to the wild-type (WT) protein, whereas mutations D320A or D324A completely abolished its activity. Similarly, mutations R319A, R497A, or T504A substantially reduced the activity of LaLIMS compared to those of WT, with D356A or D360A leading to complete loss of activity. For LaBERS, mutations R254A, R432A, or T439A markedly decreased the activity compared to that of WT protein, while D291A or D295A resulted in total inactivity. The expression levels of the *LaLINS*, *LaLIMS*, and *LaBERS* genes were notably higher in leaves compared to those in the stems and flowers, peaking at 8:00 a.m. These findings provide foundational insights into the mechanisms of LaLINS, LaLIMS, and LaBERS, and may inform strategies for improving the quality of lavender essential oils through genetic engineering.

Unfortunately, we were unable to obtain crystals of LaLINS, LaLIMS, or LaBERS, which led us to pursue a more in-depth exploration of their functional mechanisms. To assist in this investigation, we used SWISS-MODEL (https://Swissmodel.expasy.org/) to identify structural homologs of LaLINS, LaLIMS, and LaBERS (**Supplementary Tables S2–S4**). We found that LaLINS exhibited amino acid sequence identities of 64.56%, 58.16%, 54.37%, 51.87%, and 45.76% with terpene synthases from *Salvia officinalis*, *Thymus vulgaris*, *Salvia fruticosa*, *Mentha spicata*, and *Citrus sinensis*, respectively (**Supplementary Table S2**). LaLIMS showed amino acid sequence identities of 65.81%, 60.29%, 55.83%, 53.06%, and 46.88% with terpene synthases from *S. officinalis*, *T. vulgaris*, *S. fruticosa*, *M. spicata*, and *C. sinensis*, respectively (**Supplementary Table S3**). For LaBERS, the amino acid sequence identities were 48.58%, 45.37%, 44.92%, 44.38%, and 42.96% with terpene synthases from *C. sinensis*, *S. fruticosa*, *M. spicata*, *Gray Poplar Leaves* (*Populus x canescens*), and *Santalum album*, respectively (**Supplementary Table S4**). These findings provided valuable insights into the structural and functional mechanisms of the three terpene synthases in lavender.

The expression of terpene synthase genes is known to be significantly upregulated in specialized cells, such as glandular trichomes, which are found on the aerial parts of plants (Tholl, 2006; Mendes et al., 2014). Many terpene synthases from the Lamiaceae family, including linalool synthase, play a role in terpenoid biosynthesis within these secretory glandular trichomes (Markus Lange and Turner, 2012). Conversely, *Actinidia arguta* linalool synthase exhibits constitutive expression with a slight decrease in the morning and an increase at midday, reaching peak linalool emission at 8:00 a.m (Chen et al., 2010). This pattern may be analogous to findings in *Pinus pinea*, where the substantial emission of oxygenated monoterpenoid linalool is regulated by stomatal opening, which is influenced by light intensity and temperature (Niinemets, 2002; Hamberger et al., 2020). A notable reduction in monoterpenoid emission during midday, attributed to diurnal water stress and closed stomata, has been observed. However, it remains unclear whether such diurnal water stress and stomatal closure can account for the terpenoid emission patterns in lavender (Niinemets, 2002; Hamberger et al., 2020).

In summary, our study offers a novel methodology for the comprehensive investigation of the intricate functional mechanisms of LaLINS, LaLIMS, and LaBERS in lavender, and aims to improve the quality of lavender essential oils.

## Materials and methods

### Protein constructs, expression, and purification

The procedures for constructing, expressing, and purifying various protein constructs of the three terpene synthases were conducted in accordance with established methods reported previously (Landmann et al., 2007; Liu et al., 2025).

### Structure prediction and quality assessment of target proteins

The two-dimensional (2D) structures of the three terpene synthases were analyzed using the Phyre2 tool (Kelley et al., 2015). The three-dimensional (3D) structure predictions of these enzymes were carried out using the AlphaFold2 program (Jumper et al., 2021; Tunyasuvunakool et al., 2021; Wayment-Steele et al., 2023). Sequences for the terpene synthases were retrieved from the UniProt database (LaLINS, entry ID Q2XSC5; LaLIMS, entry ID Q2XSC6; LaBERS, entry ID Q2XSC4). Structural visualizations were generated using PyMOL 2.3.4 (https://www.pymol.org/2/). The quality of the structural model was evaluated through protein tertiary structure visualization and conformation charts produced by Ramachandran Plot analysis using PDBsum.

### Dynamic light scattering experiments

To investigate the oligomeric state of the three terpene synthases, their diameters were determined using dynamic light scattering (DLS) with a Dynapro DLS instrument (Malvern Zetasizer, Malvern, UK) following slight modifications (Liu et al., 2024). Each enzyme was concentrated to approximately 1.8 mg/ml and then subjected to centrifugation at 12,000 rpm at 4°C for 5 min. The enzymes were subsequently introduced into 1-cm path length cuvettes. Data acquisition involved performing 30 runs per sample, with an equilibration period of 120 s. The DLS data were analyzed using Zetasizer software (Version 6.20), and regularized DLS histograms were generated. The diameter of the particles was continuously monitored throughout the analysis.

### Enzymatic activity assays for wild-type three terpene synthases

The activities of the three terpene synthases were measured using a previously described method with some modification (Landmann et al., 2007). Standard assays were conducted in a total reaction volume of 500 μl, which included a buffer solution (25 mM Tris-Cl, pH 7.5, 5% glycerol, 1 mM DTT), 50 μM substrate (geranyl, farnesyl, or geranylgeranyl diphosphate), and 2–20 μg of purified recombinant enzyme. The reaction mixture was layered with 500 μl of diethyl ether and incubated at 23°C for a duration of 10 to 15 min under optimal conditions (Landmann et al., 2007). The reaction was terminated by vigorous mixing followed by centrifugation to facilitate phase separation. An internal standard was subsequently introduced (1 μg camphor for LaLIMS and LaBERS, and 0.164 μg [1,2-^2^H_2_]-linalool for LaLINS), and the upper solvent phase was collected. A second extraction with an additional 500 μl of diethyl ether was performed. The combined extracts were concentrated to approximately 300 μl under a nitrogen stream, dried with sodium sulfate (Na_2_SO_4_), and subsequently analyzed using GC-MS (gas chromatography-mass spectrometry).

### GC-MS analysis

GC-MS (gas chromatography-mass spectrometry) analysis was conducted using an Agilent GC 6850 gas chromatograph coupled with an Agilent 5973 ion trap mass detector following a previously established method with some modifications (Landmann et al., 2007). The system was equipped with a 30 m × 0.25 mm apolar capillary column (DB5). The injector and detector temperatures were maintained at 250°C. Helium served as the carrier gas, delivered at a flow rate of 1.0 ml/min. The oven temperature program involved an initial hold at 60°C for 4 min, followed by a temperature ramp of 4°C/min until reaching 240°C, where it was held for an additional 5 min. A 2-μl injection was performed in splitless mode. Molecular identification was achieved using mass spectra databases, including Wiley, NIST 05, and Adams. The identification of linalool, limonene, and alpha-bergamotene was based on comparisons of the GC-MS data with those of authentic reference compounds.

### Enzymatic activity assays for site-directed mutagenesis of the three terpene synthases

Primers for site-directed mutagenesis of the target proteins were designed by our lab and synthesized by Shanghai Sangon Biotechnology (China) (**Supplementary Tables S5–S7** and **Supplementary Figures S6–S8**). Q5 polymerase from NEB (New England Biolabs) was used for PCR. Sequencing of the DNA was conducted by Shanghai Sangon Biotechnology (Shanghai, China) to confirm the accuracy of cloning sites. The procedures for expressing and purifying the mutated terpene synthases were identical to those used for the wild-type (WT) protein (Landmann et al., 2007). Additionally, the enzymatic activities of the mutated terpene synthases were evaluated under conditions equivalent to those used for the WT protein.

### RT-qPCR analyses of expression levels of three terpene synthases

To quantify the expression levels of the three terpene synthases, real-time quantitative polymerase chain reaction (RT-qPCR) was performed using PowerUp SYBR Green Master Mix (Applied Biosystems). Total RNA was extracted with the Universal Plant Total RNA Extraction Kit (Bioteke, Beijing, China), following the manufacturer’s instructions. cDNA was subsequently synthesized from the RNA samples using the PrimeScript 1st Strand cDNA Synthesis Kit (Takara, Kyoto, Japan). Primers used for the analysis are detailed in **Supplementary Table S1**. RT-qPCR was conducted with an Applied Biosystems QuantStudio 5 instrument. Data analysis was performed using the 2^−ΔΔCT^ method (Rocha et al., 2015; Biassoni and Raso, 2022; Hajeri et al., 2022; Liu et al., 2024), with relative expression ratios presented as log_2_ values in histograms. Gene beta-actin (entry ID A0A2I8B2D2) served as the housekeeping gene for data normalization (**Supplementary Figure S9**), and a positive control using the beta-actin gene was included in the analysis.

### Statistical analysis

All experiments were conducted at least in triplicate. The data were expressed as mean ± SD. Statistical analysis was conducted using Origin 8.5, Microsoft Excel 2013, and SPSS 19.0. In the all statistical evaluations, p < 0.05 was considered statistically significant, and p < 0.01 was considered high statistically significant.

## Data Availability

The original contributions presented in the study are publicly available. This data can be found here: Harvard Dataverse, https://doi.org/10.7910/DVN/7OSIGG, accession 0f97db29835c1e88ec91b699e69ce8e6.
