# Supplementary material for: Insights into the functional mechanisms of three terpene synthases from Lavandula angustifolia (Lavender)
Source: Front Plant Sci. 2024 Dec 3;15:1497345. doi: 10.3389/fpls.2024.1497345 (PMC11649398; doi:10.3389/fpls.2024.1497345)
Supplement: Supplementary file 1 [file DataSheet1.pdf]

Supplementary Material

Insights into the functional mechanisms of three terpene synthases from *Lavandula angustifolia* (Lavender)

Dafeng Liu[1,2,*], Hongying Song[2], Huashui Deng[2], Ablikim Abdiriyim[1], Lvxia Zhang[1], Ziwei Jiao[1], Xueru Li[1], Lu Liu[1] and Shuangqin Bai[1]

[1]Xinjiang Key Laboratory of Lavender Conservation and Utilization, College of Biological Sciences and Technology, Yili Normal University, Yining 835000, Xinjiang, China;

[2]School of Life Sciences, Xiamen University, Xiamen 361102, Fujian, China.

[*]Correspondence: dafeli@sina.cn or liudafeng2017@163.com

CONTENTS

Table S1. .... S3
Table S2. .... S4
Table S3. .... S5
Table S4. .... S6
Table S5. .... S7
Table S6. .... S8
Table S7. .... S9
Figure S1. .... S10
Figure S2. .... S12
Figure S3. .... S13
Figure S4. .... S14
Figure S5. .... S15
Figure S6. .... S16
Figure S7. .... S17
Figure S8. .... S18
Figure S9. .... S19

Table S1.

Table S1. Primers used for RT-qPCR in this study

Genes	Primers	Primer sequence (5'-3')
Beta-actin	Forward primer	ggcagtttggacaagagaagacacagtcacc
	Reverse primer	ttttttgattaaaaaaaaaaagctgaaatcataatatttttaat
LaLINS	Forward primer	atgtcgatcaatatcaacatgcctgcagcc
	Reverse primer	tcatgcgtacggctcgaacagcatg
LaLIMS	Forward primer	atgtctatcattagcatgcatgtgggaatccttaataggc
	Reverse primer	ttagggatatggctcgaacatcagggttcca
LaBERS	Forward primer	atggaggcgagaaggtcgggg
	Reverse primer	tcatggcatatggaagggtagaacaatgagtgacac

Table S2.

Table S2. Search for structural homologs of LaLINS using SWISS-MODEL

Rank	PDB code	GMQE	Identity (%)	Method	Oligo State	Ligands	Description
1	1n1z	0.87	64.56	X-ray, 2.3 Å	homo-dimer	6xMG, 2xBTB, 2xPOP	(+)-bornyl diphosphate synthase; (+)-Bornyl Diphosphate Synthase: Complex with Mg and pyrophosphate from *Salvia officinalis*
2	5c05	0.82	58.16	X-ray, 1.6 Å	homo-dimer	None	Putative gamma-terpinene synthase; Crystal Structure of Gamma-terpinene Synthase from *Thymus vulgaris*
3	2j5c	0.74	54.37	X-ray, 2.0 Å	monomer	None	1,8-CINEOLE SYNTHASE; Rational conversion of substrate and product specificity in a monoterpene synthase. Structural insights into the molecular basis of rapid evolution from *Salvia fruticosa*
4	2onh	0.76	51.87	X-ray, 2.7 Å	homo-dimer	6xMN, 2xFPG, 4xBTB	4S-limonene synthase; Crystal Structure of limonene synthase with 2-fluorogeranyl diphosphate (FGPP) from *Mentha spicata*
5	5uv0	0.76	45.76	X-ray, 2.3 Å	monomer	None	(+)-limonene synthase; Crystal Structure of (+)-Limonene Synthase from *Citrus sinensis*

Table S3.

Table S3. Search for structural homologs of LaLIMS using SWISS-MODEL

Rank	PDB code	GMQE	Identity (%)	Method	Oligo State	Ligands	Description
1	1n1z	0.84	65.81	X-ray, 2.3 Å	homo-dimer	6xMG, 2xBTB, 2xPOP	(+)-bornyl diphosphate synthase; (+)-Bornyl Diphosphate Synthase: Complex with Mg and pyrophosphate from *Salvia officinalis*
2	5c05	0.79	60.29	X-ray, 1.6 Å	homo-dimer	None	Putative gamma-terpinene synthase; Crystal Structure of Gamma-terpinene Synthase from *Thymus vulgaris*
3	2j5c	0.72	55.83	X-ray, 2.0 Å	monomer	None	1,8-CINEOLE SYNTHASE; Rational conversion of substrate and product specificity in a monoterpene synthase. Structural insights into the molecular basis of rapid evolution from *Salvia fruticosa*
4	2onh	0.74	53.06	X-ray, 2.7 Å	homo-dimer	6xMN, 2xF3P, 1xBTB	4S-limonene synthase; Crystal Structure of limonene synthase with 2-fluorolinalyl diphosphate(FLPP) from *Mentha spicata*
5	5uv0	0.72	46.88	X-ray, 2.3 Å	monomer	None	(+)-limonene synthase; Crystal Structure of (+)-Limonene Synthase from *Citrus sinensis*

Table S4.

Table S4. Search for structural homologs of LaBERS using SWISS-MODEL

Rank	PDB code	GMQE	Identity (%)	Method	Oligo State	Ligands	Description
1	5uv0	0.81	48.58	X-ray, 2.3 Å	monomer	None	(+)-limonene synthase Crystal Structure of (+)-Limonene Synthase from *Citrus sinensis*
2	2j5c	0.75	45.37	X-ray, 2.0 Å	monomer	None	1,8-CINEOLE SYNTHASE Rational conversion of substrate and product specificity in a monoterpene synthase. Structural insights into the molecular basis of rapid evolution from *Salvia fruticosa*
3	2onh	0.78	44.92	X-ray, 2.7 Å	homo-dimer	6xMN, 2xF3P, 1xBTB	4S-limonene synthase; Crystal Structure of limonene synthase with 2-fluorolinalyl diphosphate(FLPP) from *Mentha spicata*
4	3n0f	0.79	44.38	X-ray, 2.7 Å	monomer	None	Isoprene synthase; Crystal Structure of Isoprene Synthase from *Grey Poplar Leaves* (*Populus x canescens*)
5	6o9p	0.77	42.96	X-ray, 2.1 Å	monomer	1xBFQ, 2xMG	Sesquisabinene B synthase 1; Wild-type SaSQS1 Complexed with Ibandronate from *Santalum album*

Table S5.

Table S5. Primers used for generating site-directed mutants of LaLINS

Primers	Primer sequence (5′-3′)
R283A (F)	catttgtg**GCA**gataggcttgtggaaagctacttttg
R283A (R)	gcctatc**TGC**cacaaatgggagtttttcagctagg
D320A (F)	catcttta**GCA**gatgtttacgatatctatggcacgttag
D320A (R)	gtaaacatc**TGC**taaagatgtgattagggttatgatcttggt
D324A (F)	gatgtttac**GCA**atctatggcacgttagatgaattg
D324A (R)	ccatagat**TGC**gtaaacatcgtctaaagatgtgattaggg
R461A (F)	gctcgtg**GCA**cttcccgatgacctaggaaca
R461A (R)	cgggaag**TGC**cacgagcattcccgaaac
T468A (F)	cctagga**GCA**tcatcgttcgagatggagag
T468A (R)	cgatga**TGC**tcctaggtcatcgggaagc

Note: Mutagenic regions of the sequence are shown in underlined and bold.

Table S6.

Table S6. Primers used for generating site-directed mutants of LaLIMS

Primers	Primer sequence (5′-3′)
R319A (F)	cattcgcg**GCA**gatcgggttgttgagtcc
R319A (R)	cccgatc**TGC**cgcgaatgggagtttttc
D356A (F)	gttgta**GCA**gatgtttacgatgtatatggtacg
D356A (R)	gtaaacatc**TGC**tacaactgttgctagagcaataatc
D360A (F)	gtttac**GCA**gtatatggtacgttagaggaactg
D360A (R)	ccatatac**TGC**gtaaacatcatctacaactgttgc
R497A (F)	gcttcta**GCA**cttcctgatgatctaggaacag
R497A (R)	caggaag**TGC**tagaagcattcctgagagg
T504A (F)	gatctagga**GCA**gcatcgtttgagttgaagag
T504A (R)	cgatgc**TGC**tcctagatcatcaggaagcc

Note: Mutagenic regions of the sequence are shown in underlined and bold.

Table S7.

Table S7. Primers used for generating site-directed mutants of LaBERS

Primers	Primer sequence (5'-3')
R254A (F)	gttttgcc**GCA**caccgattggcagag
R254A (R)	caatcggtg**TGC**ggcaaaactcatcttttcg
D291A (F)	cgataaca**GCA**gacatatacgacatttatggagc
D291A (R)	gtatatgtc**TGC**tgttatcgtaattagcactgcaatc
D295A (F)	catatac**GCA**atttatggagcgttagaagaac
D295A (R)	ccataaat**TGC**gtatatgtcatctgttatcgtaattag
R432A (F)	ggttctt**GCA**cttgcagatgatttgggaac
R432A (R)	ctgcaag**TGC**aagaaccatggatggccaac
T439A (F)	gatttggga**GCA**tcatcagaagaaatcaaaagagg
T439A (R)	ctgatga**TGC**tcccaaatcatctgcaag

Note: Mutagenic regions of the sequence are shown in underlined and bold.

Figure S1.

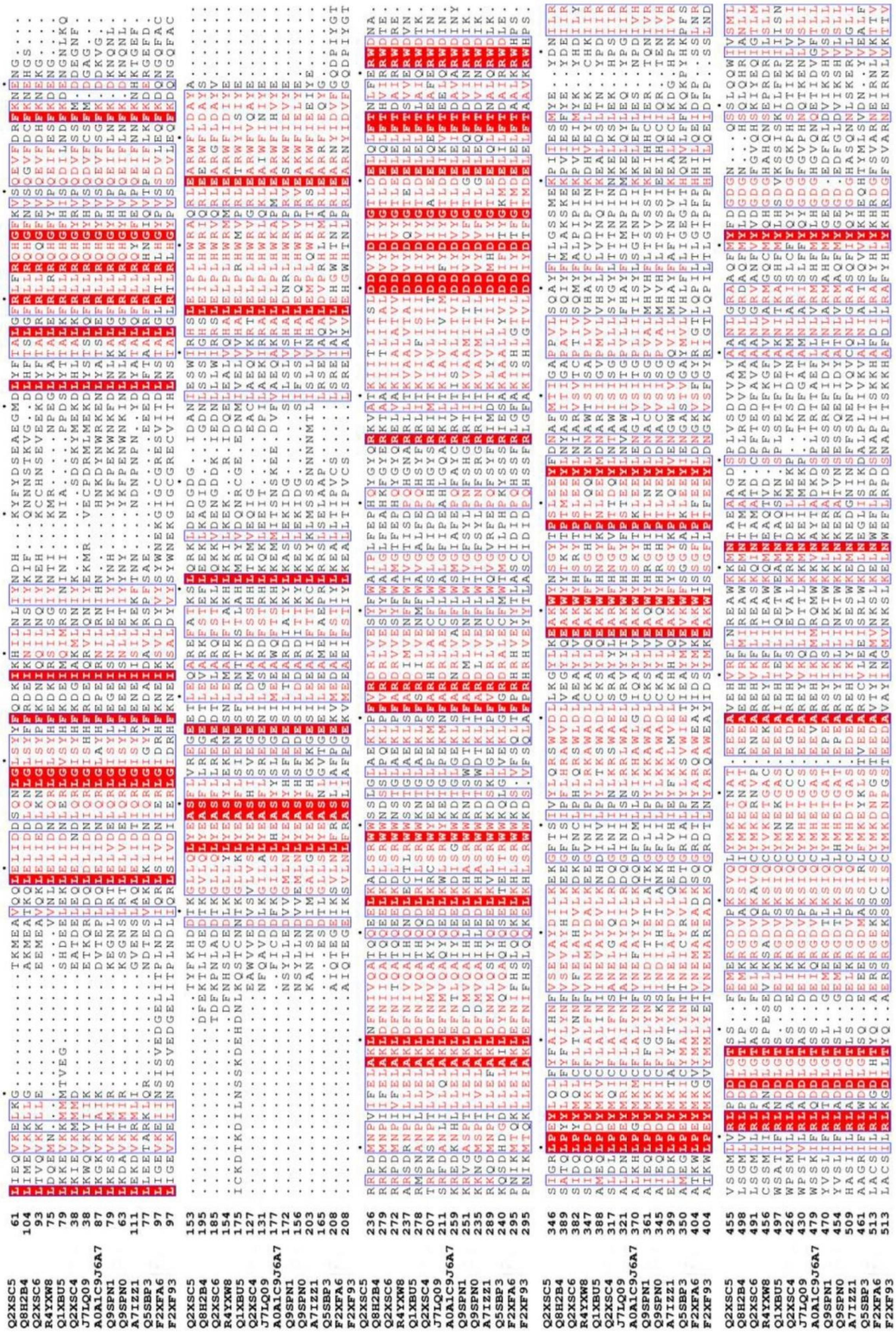

Figure S1. Sequence alignments of terpene synthases. The ClustalW default color scheme is employed, wherein conserved amino acids are depicted with more intense colors compared to non-conserved ones. The alignment includes the following reference proteins: Q2XSC5, *Lavandula angustifolia* (Lavender); Q8H2B4, *Mentha aquatica* (Water mint); Q2XSC6, *Lavandula angustifolia* (Lavender); R4YXW8, *Coffea arabica* (Arabian coffee); Q1XBU5, *Solanum lycopersicum* (Tomato, Lycopersicon esculentum); Q2XSC4, *Lavandula angustifolia* (Lavender); J7LQ09, *Phyla dulcis* (Aztec sweet herb, Lippia dulcis); A0A1C9J6A7, *Citrus sinensis* (Sweet orange, Citrus aurantium var. sinensis); Q9SPN1, *Artemisia annua* (Sweet wormwood); Q9SPN0, *Artemisia annua* (Sweet wormwood); A7IZZ1, *Cannabis sativa* (Hemp, Marijuana); Q5SBP3, *Ocimum basilicum* (Sweet basil); F2XFA6, *Picea sitchensis* (Sitka spruce, Pinus sitchensis); F2XF93, *Picea glauca* (White spruce, Pinus glauca).

Figure S2.

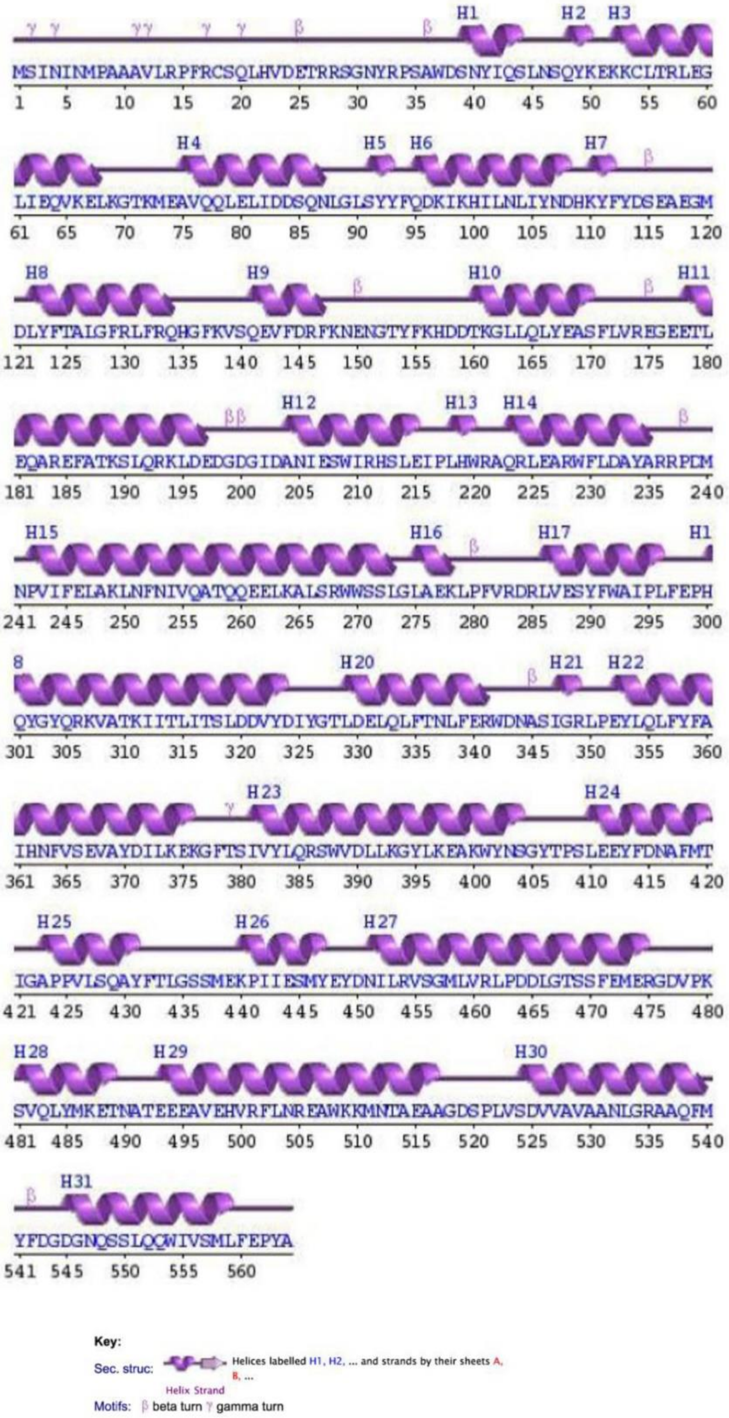

Figure S2. Secondary structure of LaLINS.

Figure S3.

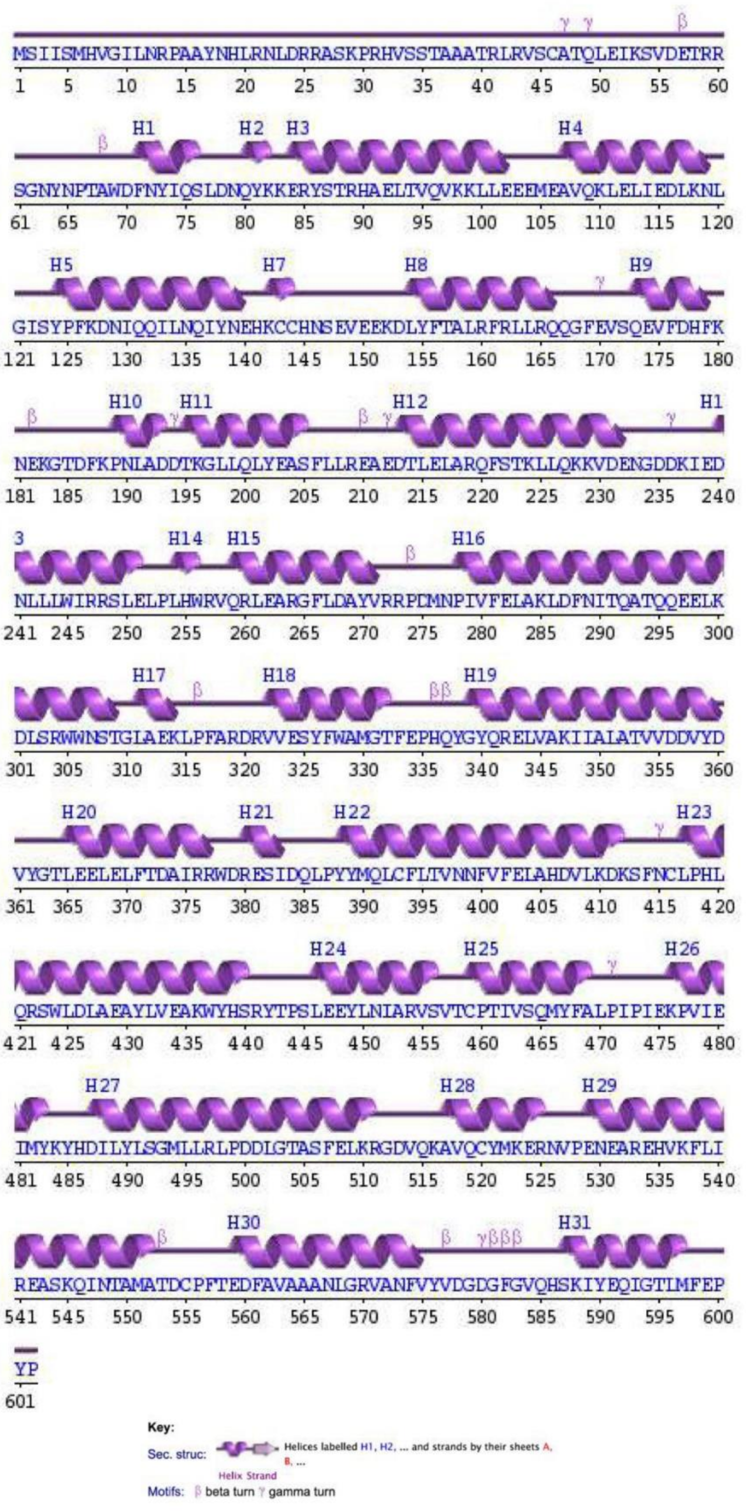

Figure S3. Secondary structure of LaLIMS.

Figure S4.

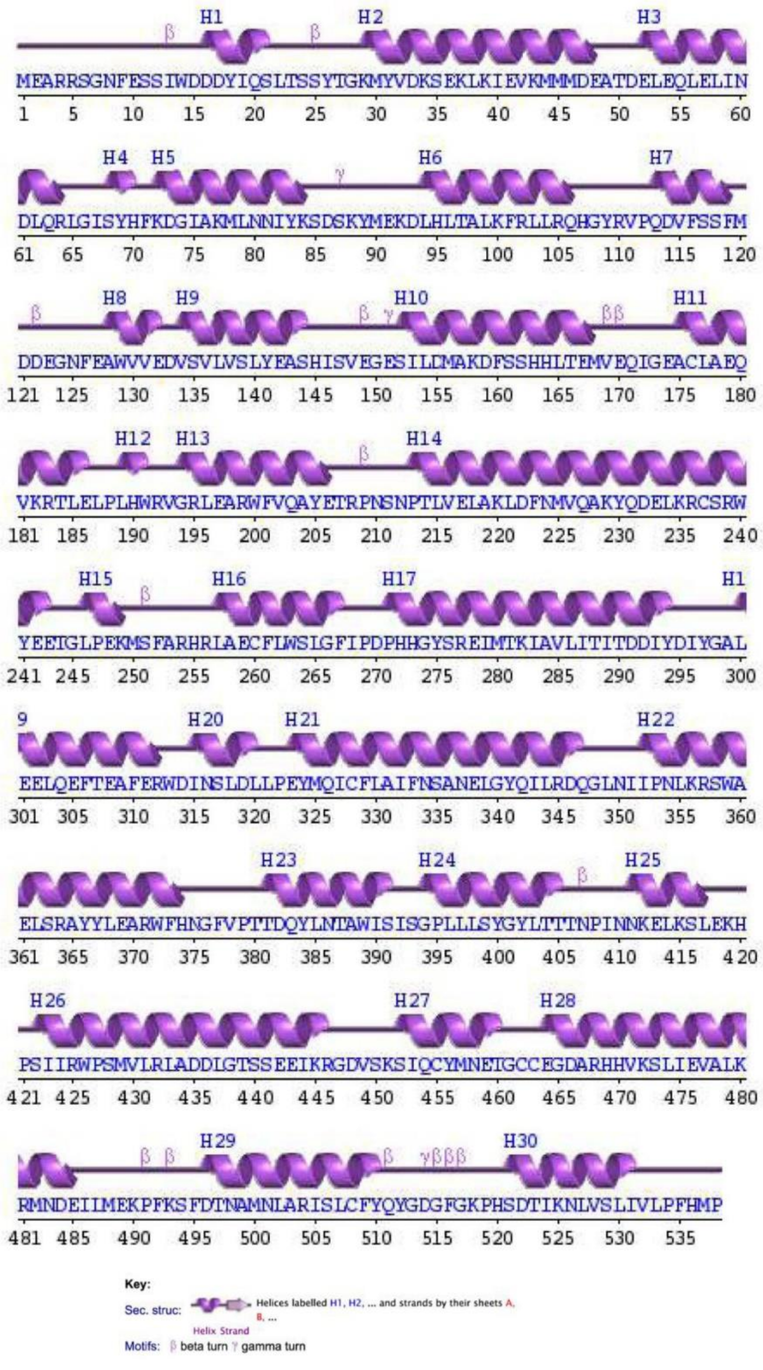

Figure S4. Secondary structure of LaBERS.

Figure S5.

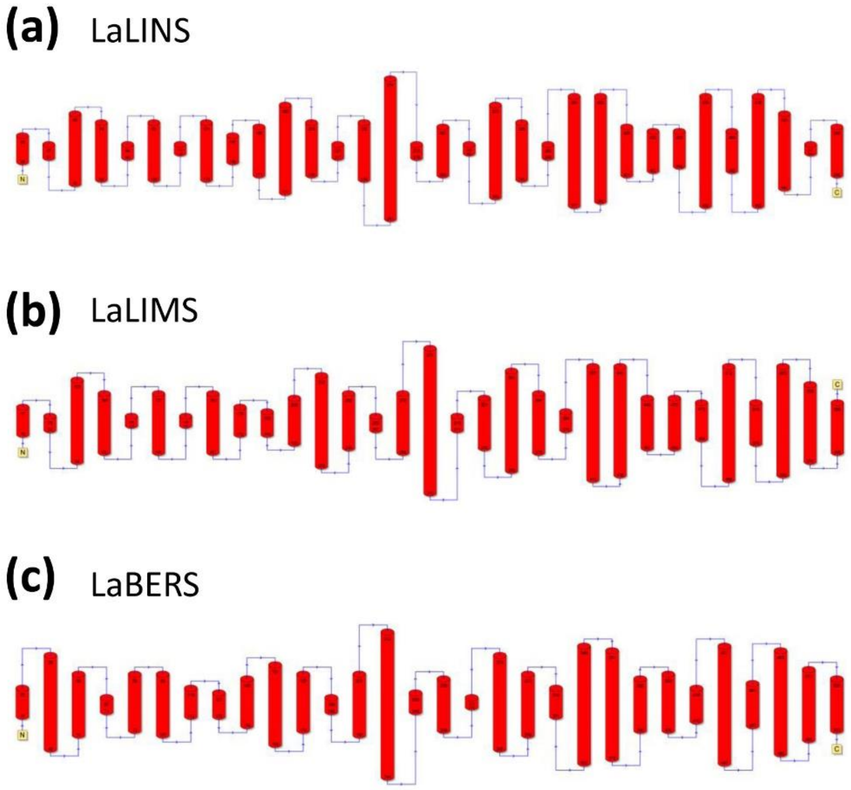

Figure S5. Secondary structure of the three terpene synthases.

Figure S6.

atgtcgatcaatatcaacatgcctgcagccgccgtcctccgcccttttcgctgctcacaactacatgtcgatgaaacccgacgctccggaaact
accgcccctcggcttgggattccaactacatccaatctctcaattctcagtataaggaaaagaagtgcttgacaaggctagaagggctgattg
agcaagtgaaggaactgaaggggacaaaaatggaggctgttcaacaattggagttgattgatgactcgcagaatctgggattatcatattat
tttcaagataaaattaaacatatcttgaatttgatatataatgatcacaaatatttttacgatagtgaagctgaaggaatggatttgtattttaca
gctcttggatttagactctttagacaacatggttttaaagtctcccaagaagtatttgatcgtttcaagaacgagaatggtacgtatttcaagca
cgacgatacaaagggattgttgcagctctacgaagcatcattcctagtgcgagaaggcgaagagacactcgaacaagcacgagaatttgcc
accaaatccctacaaagaaaacttgatgaggatggtgatggaattgacgccaatatcgaatcatggatccgccactctctggagatcccact
tcattggagggctcagaggctagaggcgagatggttcctagatgcttatgcgagaaggcccgacatgaaccccgttatcttcgagcttgctaa
actcaacttcaatattgtccaagcaacacaacaagaagaattgaaagctctctcgaggtggtggagtagtttaggcctagctgaaaaactcc
catttgtgagggataggcttgtggaaagctacttttgggctattccactctttgagcctcatcaatatggatatcaaagaaaagtggccaccaa
gatcataaccctaatcacatctttagacgatgtttacgatatctatggcacgttagatgaattgcaactatttacgaacttatttgaaagatggg
ataatgcatcaatcggccgacttcctgaatacttgcaattgttctatttcgcaatccacaactttgtttccgaggtggcttacgacattctcaaag
aaaagggtttcactagtattgtatatttacagagatcgtgggtggatttgctaaaaggatacctaaaagaggcaaagtggtacaatagtgga
tacacgccaagcctcgaggaatatttcgacaacgcattcatgacaataggggcccctccggtactatcgcaagcttatttcacattaggaagc
tcgatggagaaaccgatcatcgagagcatgtacgaatatgacaacatacttcgcgtttcgggaatgctcgtgaggcttcccgatgacctagg
aacatcatcgttcgagatggagagaggcgacgtgccgaaatcggtccagctatacatgaaggaaacaaatgctacggaggaggaggcggt
ggagcacgtgaggtttttgaatcgggaggcgtggaagaagatgaacacggcggaggcggccggtgattctccgttagtgagtgacgtggtg
gcggtggcggcgaatcttggaagggcggcgcagtttatgtatttcgacggagatggtaaccagtctagtttgcagcagtggattgtgagcatg
ctgttcgagccgtacgcatga

Figure S6. Gene sequence of *LaLINS*

Figure S7.

atgtctatcattagcatgcatgtgggaatccttaataggcctgcagcttataaccatcttcgcaacttggacaggagagcttcaaagccgcgcc
atgtctcttctactgccgccgccactcgcctccgggtttcttgcgccacacaactagaaattaagtccgtcgatgaaacccgacgctccggaaa
ctacaaccctaccgcttgggatttcaactacatccaatccctcgacaatcagtataagaaagagaggtactcgacaagacacgctgagctga
ctgtgcaagtgaagaagctgctggaggaagaaatggaagcggttcaaaagttggaattgattgaggatttgaagaacctgggaatatctta
cccatttaaggacaatatccaacagattttaaatcaaatatataatgagcacaaatgttgccacaacagtgaagtggaagaaaaggatttgt
atttcacggctcttcgattccgactccttagacaacagggttttgaagtctctcaagaagtatttgatcatttcaagaacgagaagggtacaga
tttcaagccaaaccttgctgacgatactaaaggactattgcaactttacgaagcatctttcctattaagagaagctgaagatacacttgagtta
gctcgacaattttcaaccaaattactgcagaaaaaagtcgatgagaatggtgatgataaaatagaggataatctattattatggattcgccgt
tctttggagctcccgcttcattggagggtgcaaaggctagaagcaagagggttcttggatgcttacgttagaaggcccgacatgaatccaatt
gtttttgagctcgccaaactcgacttcaatattacccaagcaacacaacaagaagaactgaaagatctctcgaggtggtggaatagtacagg
ccttgccgaaaaactcccattcgcgagggatcgggttgttgagtcctacttctgggcaatgggaacctttgagcctcatcaatatggttatcag
agagaacttgtcgccaagattattgctctagcaacagttgtagatgatgtttacgatgtatatggtacgttagaggaactggaactatttacag
atgccattcggagatgggatcgtgaatcaatcgaccaacttccttactacatgcagctatgctttttgactgtcaacaactttgttttcgagcttg
ctcatgatgttcttaaggataagagtttcaactgcttaccacatttacagagatcgtggctagacttggctgaagcatatcttgtcgaggctaag
tggtaccacagtagatatacaccgagcctcgaggaatatctcaatattgcaagagtttcagttacgtgtcccactatagtttcacaaatgtactt
tgcattaccaattccgatagagaaaccggtcatcgagatcatgtacaaataccacgacatactttacctctcaggaatgcttctaaggcttcct
gatgatctaggaacagcatcgtttgagttgaagagaggtgatgtgcaaaaagcagtccagtgttatatgaaggaaagaaatgttcctgaaaa
tgaggcacgagaacatgtgaagtttctgattcgggaggcgtcgaagcagataaacaccgcgatggcgaccgattgtccatttactgaagatt
ttgctgtggctgcagcgaatcttggaagagtggcgaattttgtgtacgtcgacggagatggttttggcgtgcaacactcaaaaatatatgaac
agattggaaccctgatgttcgagccatatccctaa

Figure S7. Gene sequence of *LaLIMS*

Figure S8.

atggaggcgagaaggtcggggaatttcgagtccagcatctgggacgacgattacattcagtccctaacgagttcatatacgggtaaaatgta
cgtagataaatctgagaagctgaaaattgaggtgaagatgatgatggacgaagcaacagatgagttggagcaactcgagcttatcaacgat
ctgcaaaggcttggtatatcttaccacttcaaagatggcatagctaagatgttgaacaacatttataagtcagatagcaaatacatggaaaa
ggacttgcacctcacagctctcaaatttagacttcttagacaacatggatatcgtgttcctcaagatgtttttagtagtttcatggatgatgaagg
aaatttcgaggcctgggtggttgaagacgtgtcagttcttgtatctctgtatgaagcttcgcacatatcagtggaaggggagagcatattggat
atggcgaaagatttctcatcgcatcatcttactgaaatggtggagcagattggtgaggcgtgtttagccgagcaagtgaagcgtacgttggag
cttccactacactggagagttgggagacttgaagccagatggttcgtacaagcctacgagactagacccaactcaaaccccactctggtgga
gctagccaaactagatttcaacatggtacaagccaaataccaggatgagcttaaacgctgttcaaggtggtatgaagaaactggcctaccc
gaaaagatgagttttgccagacaccgattggcagagtgtttttgtggtctctgggatttataccagatcctcatcatgggtattcgagggagat
tatgaccaagattgcagtgctaattacgataacagatgacatatacgacatttatggagcgttagaagaacttcaagagttcacagaggcatt
cgagagatgggatatcaattcattggatctccttccagaatacatgcaaatctgtttcttagcaatcttcaactctgcaaatgaattgggttatc
aaattctgagggaccaaggcctcaatattatcccaaatctgaagagatcgtgggcagaattgagtagagcttactacttagaagcaagatgg
tttcacaacggatttgtcccaactacagatcagtatctgaatactgcttggatttccatttcgggtcctctacttctttcctatggctacctcacca
ccacgaatcccataaacaacaaggaactgaagagcctagaaaaacatcctagtatcatccgttggccatccatggttcttcgacttgcagat
gatttgggaacttcatcagaagaaatcaaaagaggggatgtctcgaaatcaatccaatgctacatgaatgaaacaggatgctgcgagggg
gatgcccgacatcacgtaaagagtttgattgaggtagcattgaagcgaatgaacgatgagatattgatggagaagcccttcaagagtttcga
caccaacgccatgaatcttgcacgaatatctctctgcttctatcaatatggtgatggtttcgggaagccccactctgataccatcaagaacttg
gtgtcactcattgttctacccttccatatgccatga

Figure S8. Gene sequence of *LaBERS*

Figure S9.

ggcagtttggacaagagaagacacagtcacccattttctgcaagtcaagaacgcattaacaaatggccgaagccgaggatattcagcccctt
gtctgtgacaatggaactggaatggtcaaggctggatttgctggagatgatgctccaagggccgtcttccctagcattgttgggcgccctcgtc
acactggtgtgatggttggcatgggccaaaaagatgcttatgttggtgacgaggctcagtccaagagaggtatattgacattgaagtatccga
ttgagcacggaattgtcagcaactgggatgatatggagaaaatatggcatcacacattctacaatgagcttcgtgtagccccagaagagcac
ccgattctcttgacagaggctcctcttaatcccaaggccaatcgtgagaagatgactcagatcatgtttgagacctttaatgcccctgccatgta
tgttgctattcaggcagttctctcactctatgccagtggtcgtacaactggtattgttctggactctggtgatggtgtgagtcacacagtccccat
ctacgaaggttatgcactccctcatgcaatccttcgtcttgatctggctgggcgtgatctcaccgactccctcatgaagatattgaccgagaga
ggctactcattcacaaccactgctgagcgggaaattgtgagggacatcaaagagaagctggcttacattgcactggactacgaacaggagc
tggagacagcaaagaccagctccgctgtggagaagaactacgaactgcctgatggacaggtcatcactatcggtgctgagcggttccgctg
ccctgaggttctgttccagccatcaatgatcggaatggaagctgctggaatccacgagaccacttacaattccatcatgaagtgtgatgtcgat
atcagaaaggatctgtacggaaacattgtcctcagtggtgggtcgactatgttccccggtattgctgataggatgagcaaggagatcaccgct
ctggctcccagcagcatgaagattaaggtggtggctccgcccgagaggaagtactctgtctggataggaggttccattttggcttctctcagc
accttccagcagatgtggattgccaaggcagagtacgacgaatctggcccgtcgatcgttcacagaaaatgcttctaaggaggagagcttgg
gtgaagagagatttgccctgctgttgctgcctgctcatttggtttctgttttagtttctggtgttattttactcctctcctatgagcttttactttttaa
gatttgatgaagctttggacatggtgacagacaatattcatttttccaatttgtagaagagtttgtgtattaaaaatattatgatttcagctttttttt
tttaatcaaaaaa

Figure S9. Sequence of housekeeping gene beta-actin
